# Percutaneous embolization of sporadic lumbar nerve root haemangioblastoma under local anaesthesia

**DOI:** 10.1259/bjrcr.20190037

**Published:** 2020-02-12

**Authors:** Ho Sang Leung, Ryan Ka Lok Lee, Eric Ka Chai Law, Wai Kit Mak, James Francis Griffith, Simon Chun Ho Yu

**Affiliations:** 1 Department of Imaging and Interventional Radiology, Prince of Wales Hospital, Hong Kong, China; 2 Division of Neurosurgery, Department of Surgery, Prince of Wales Hospital, Hong Kong, China

## Abstract

Pre-operative embolization of spinal tumours are mainly performed using a transarterial approach. Percutaneous embolization of spinal tumours are undertaken much less frequently, though its use has been reported in hypervascular spinal metastases^1,2^ and spinal paraganglioma.^3^ We present a patient in whom pre-operative percutaneous embolization has been performed to a recurrent lumbar nerve root haemangioblastoma that had previously been embolized using a transarterial approach. Percutaneous embolization, through targeted percutaneous puncture of the extradural component, helped reduce intraoperative blood loss, and minimize risk of spinal ischaemia.

## Clinical presentation

A 59-year-old male presented with low back pain to a general practitioner. Initial screening blood tests revealed polycythaemia (haemoglobin = 24.1 g dl^−1^). PET-CT and MRI were performed, which showed a mass within the right psoas muscle at the L4 level with intradural extension through the right L3/4 and L4/5 foramina. There were no features of von Hippel-Lindau syndrome present clinically or radiologically. He was subsequently referred to the neurosurgical clinic. CT-guided biopsy revealed a highly vascularized haemangioblastoma. Tumour excision was planned following transarterial embolization of the feeding vessels. Transarterial embolization involved embolizing the tributaries arising from the L3 and L4 lumbar arteries using coils and particles. The day after transarterial embolization, subtotal tumour excision, sparing the component of the tumour which involved the right lumbosacral plexus to avoid nerve injury, was performed under general anaesthesia. Total blood loss during the operation was 3000 ml.

**Figure 1.**
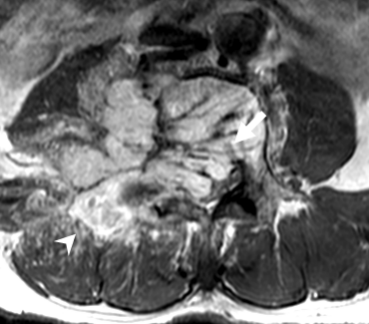
Pre-percutaneous embolization. Axial *T*_1_ weighted contrast-enhanced MRI of the lumbar spine showing the haemangioblastoma. Significant intraspinal tumour extension is present (solid arrow). An avidly enhancing extradural component is also noted at the right posterior aspect of the tumour (arrowhead).

He was followed up in the neurosurgical clinic with subsequent serial MRIs showing a gradual increase in the size of the tumour. He suffered from worsening symptoms of lower limb numbness and claudication 3.5 years after the initial operation. Another further MRI examination (Figure 1) revealed further enlargement of the tumour with extension into the spinal canal at the L4 level, resorption of the L4 pedicles and resultant spinal canal stenosis.

## Treatment

After discussion with the patient, a second operation was planned, the main aim of which was decompression of the central spinal canal and symptom relief. However, significant blood loss was anticipated during this operation. Pre-operative fluoroscopic-guided embolization of the extradural component was therefore performed with the aim of reducing blood loss during exploration and dissection of the extradural component, which was necessary for satisfactory intraoperative exposure and debulking of the central spinal canal component.

**Figure 2.**
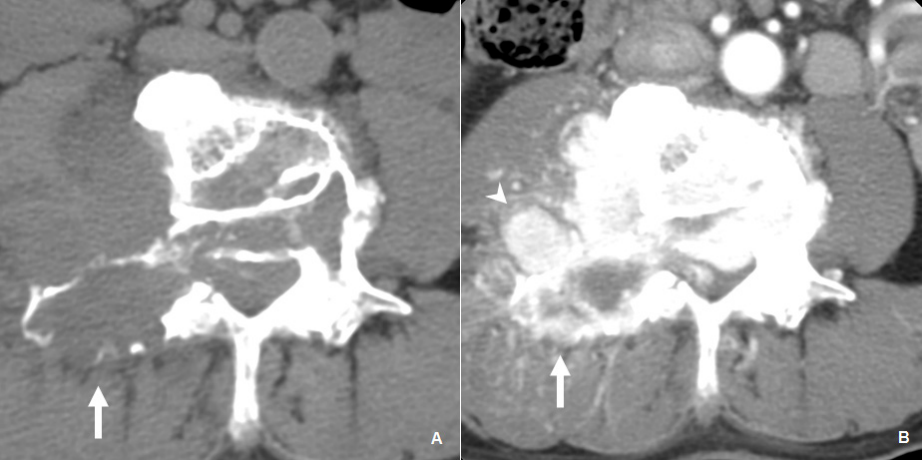
Pre (left) and arterial phase (right) axial CT images showing the recurrent haemangioblastoma with intradural and osseous extension. Arterial contrast enhancement is present in the hypervascular extradural components (arrowhead). There is bony erosion of the right pedicle and transverse process by the hypervascular tumour (solid arrow).

**Figure 3.**
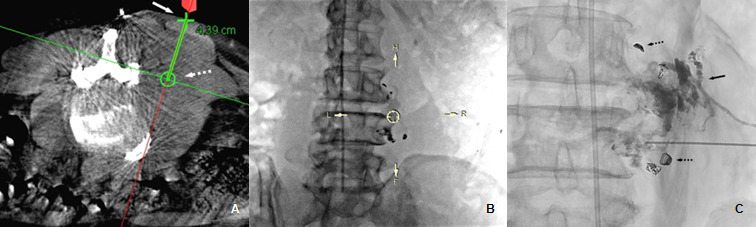
Percutaneous embolization of right L4 haemangioblastoma. A. Pre-procedural planning of needle trajectory, showing the needle entry site (white solid arrow) and planned target (white dashed arrow). B. Bulls-eye view of needle guidance by syngo iGuide technique. C. Fluoroscopic image during the needle advancement showing injection of glue into extradural component, with satisfactory lipiodol staining of the extradural component of the tumour (black solid arrow). Minor adjustments have subsequently been made to allow in-profile alignment with the Bulls-eye view as shown in Figure 4B. Embolization coils from prior trans-arterial embolization are noted (black dashed arrows).

Fluoroscopic-guided percutaneous tumour embolization was performed, as prior transarterial embolization technically prevented further trans-arterial embolization. Pre-procedural contrast-enhanced CT (Figure 2) was performed to map the vascular components of the tumour. This pre-operative CT demonstrated complete embolization of the lumbar artery and therefore repeat transarterial embolization was not attempted. Under local anaesthesia and fluoroscopic guidance, percutaneous embolization of the vascular extraspinal component of the tumour was performed using a syngo iGuide^TM^ needle guidance system (Siemens, Munich, Germany) (Figure 3). Glue embolization of the extradural tumour component was performed with diluted N-butyl cyanoacrylate (suspended in lipiodol using a dilution ratio of 1:1 to 1:5 depending on the flow rate at the site of injection). The glue was injected using a 20G spinal needle with three passes into different parts of the tumour. A total volume equivalent to 3 ml of N-butyl cyanoacrylate was injected. As direct communication was seen between the intradural component of the tumour and the spinal arteries upon iodinated contrast injection, this intradural component of the tumour was not embolized in view of risk of inadvertent spinal artery embolization and ischaemia. Post-procedural fluoroscopy showed satisfactory lipiodol staining of the extradural tumour component. Total procedure time was 90 min, with a total DAP of 34994 μGym^2^ and total fluoroscopic screening time of 18.4 min.

Subsequent L4 laminectomy, tumour excision, and posterior spinal fixation were performed the day after percutaneous embolization. Relatively low vascularity of the embolized extradural component of the tumour was noted intraoperatively. Excision of the non-embolized intradural component was challenging due to its highly vascular nature. Total blood loss was 6000 ml, most of which occurred during excision of the non-embolized component. Satisfactory excision of the intradural component of the tumour and debulking of the extradural component was achieved. As previously, the anterolateral and extradural portion of tumour was not excised as this part of the tumour involved the lumbosacral plexus with excision carrying a high risk of nerve injury.

### Outcome and follow-up

**Figure 4.**
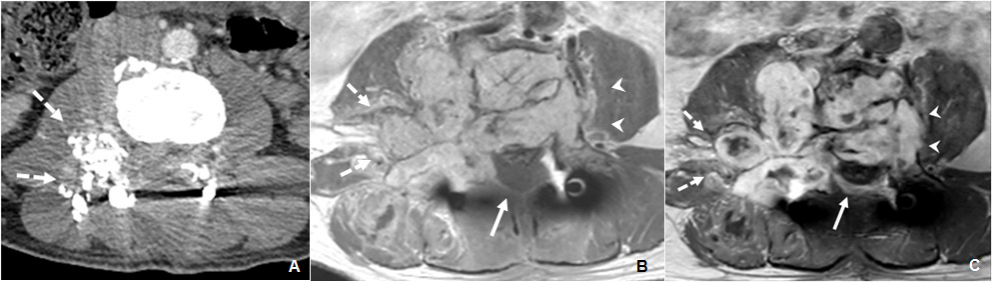
*A*. Post-procedure arterial-phase contrast-enhanced CT showing the embolized extradural component of the haemangioblastoma, with hyperdense embolization material (dashed arrows). *B*. Axial *T*_1_ weighted contrast-enhanced MRI 1 month after the percutaneous embolization and excision, showing satisfactory surgical decompression of the intradural component with reduction in spinal canal stenosis (solid arrow). Laminectomy at this level was also present with metallic surgical rods and associated susceptibility artefacts. *C*. Axial *T*_1_ weighted contrast-enhanced MRI 6 months after percutaneous embolization and excision, demonstrating satisfactory surgical decompression of the intradural component with reduction of spinal canal stenosis. Mild interval growth is present in the left paraspinal region (arrowheads), while the embolized right paraspinal component (dashed arrows) is static in size.

Post-operative CT and MRI at 1 month showed good debulking of the intradural component with mild reduction in the size of the right extradural component (Figure 4a and b). Adjuvant radiotherapy (50 Gy in 25 fractions) was given. Follow-up MRI, 6 months after the percutaneous embolization and tumour excision (Figure 4c), showed slight interval enlargement of a non-embolized component of the tumour in the left paraspinal region. The embolized right paraspinal component of the tumour was static in size, with no increase in intraspinal extension. The patient has been followed up regularly in the neurosurgical clinic for 2 years since the second operation, with minimal lower limb numbness and no motor deficit.

## Discussion

Haemangioblastoma is an uncommon benign hypervascular tumour accounting for about 2% of central nervous system tumours. About 40% of haemangioblastomas are associated with von Hippel Lindau syndrome.^4,5^ Most haemangioblastomas occur in the brainstem and spinal cord. Extramedullary nerve root haemangioblastoma is a much less common entity with about 20 reported cases.^6^ Surgical resection remains the mainstay of treatment of intra- or extramedullary haemangioblastoma, with the aim of preserving neurological function. Recurrence has been reported in up to 50% of patients following surgery.^7^ Radiosurgery is a potential alternative, especially in cases of recurrence.^3^

Pre-operative embolization using a transarterial approach has been demonstrated to be a relatively safe and efficacious method of reducing intraoperative blood loss during resection of spinal tumours. It has mainly been used to treat hypervascular metastases such as metastatic thyroid carcinoma or renal cell carcinoma.^1,2^ Embolization of a primary spinal tumour has only been reported for vertebral haemangioma,^8^ haemangiopericytoma,^9^ and cervicodorsal paraganglioma.^3^ Although considered a relatively safe procedure, transarterial embolization can be limited by the size and tortuosity of the vascular feeders. It also carries the risk of arterial reflux which may lead to inadvertent embolization of the anterior spinal artery and spinal cord infarction. Prolonged procedure time is the norm, and general anaesthesia is often required.

There has been no previous report of percutaneous embolization for the treatment of nerve root haemangioblastoma or other primary tumours in the lumbar spine. The clinical case presented herein is the subsequent clinical progress of a patient previously reported by us^10^ and demonstrates the usefulness of percutaneous embolization in primary spinal tumours in patients with prior transarterial embolization. A direct percutaneous approach gives easier access to the vascular tumour, and is particularly useful in cases where vascular access is limited following previous transarterial embolization as in this case, or with small and tortuous feeders where transarterial catheterization is difficult or not technically feasible. The syngo iGuide needle guidance system allows a three-dimensional needle path planning using cone beam CT images. Overlay of real time cone beam CT onto live fluoroscopic images is done during needle advancement, allowing for better accuracy and less needle adjustment. This seemed to facilitate easier injection into the hypervascular extradural component of the tumour, while avoiding the intradural component and minimizing the risk of inadvertent spinal artery embolization. As the procedure time is shorter and there is a less stringent requirement for patient immobility, general anaesthesia was not required. The limitations of percutaneous glue embolization include a high chance of incomplete embolization, as it does not follow the vascular supply; and a higher chance of inadvertent glue migration into the systemic veins. Percutaneous embolization is particularly useful in patients who have undergone embolization with permanent embolic materials (such as coils, particles or glue), which severely limit the feasibility of repeating transarterial embolization. Careful manipulation of glue concentration and use of higher glue concentration for the more vascularized areas of the tumour can also potentially reduce the possibility of glue migration.^11^

## Learning points

1. Extradural haemangioblastoma is an uncommon hypervascular tumour. Despite its benign features, recurrence is common and can be associated with significant neurological symptoms and morbidity.
2. Percutaneous embolization of hypervascular tumours (such as haemangioblastoma) seems an effective method to reduce tumoural vascularity and surgical blood loss, especially in patients who had undergone previous transarterial embolization.

## Declarations

1. This study was not supported by any funding.
2. The authors declare that they have no conflict of interest.
3. This article does not contain any studies with human participants or animals performed by any of the authors. Institutional Review Board (IRB) approval is waived for retrospective case reports.
4. Informed consent was obtained from the patient for publication of this case report.
